# Improving inter-observer variability in the evaluation of ultrasonographic features of polycystic ovaries

**DOI:** 10.1186/1477-7827-6-30

**Published:** 2008-07-18

**Authors:** Marla E Lujan, Donna R Chizen, Andrew K Peppin, Stefan Kriegler, David A Leswick, Terri G Bloski, Roger A Pierson

**Affiliations:** 1 Obstetrics, Gynecology & Reproductive Sciences, University of Saskatchewan, Saskatoon, Canada; 2 Radiology & Diagnostic Imaging, University of Alberta, Edmonton, Canada; 3 Academic Department of Medical Imaging, University of Saskatchewan, Saskatoon, Canada

## Abstract

**Background:**

We recently reported poor inter-observer agreement in identifying and quantifying individual ultrasonographic features of polycystic ovaries. Our objective was to determine the effect of a training workshop on reducing inter-observer variation in the ultrasonographic evaluation of polycystic ovaries.

**Methods:**

Transvaginal ultrasound recordings from thirty women with polycystic ovary syndrome (PCOS) were evaluated by three radiologists and three reproductive endocrinologists both before and after an ultrasound workshop. The following endpoints were assessed: 1) follicle number per ovary (FNPO), 2) follicle number per single cross-section (FNPS), 3) largest follicle diameter, 4) ovarian volume, 5) follicle distribution pattern and 6) presence of a corpus luteum (CL). Lin's concordance correlation coefficients (rho) and kappa statistics for multiple raters (kappa) were used to assess level of inter-observer agreement (>0.80 good, 0.60 – 0.80 moderate/fair, <0.60 poor).

**Results:**

Following the workshop, inter-observer agreement improved for the evaluation of FNPS (rho = 0.70, delta rho = +0.11), largest follicle diameter (rho = 0.77, delta rho = +0.10), ovarian volume (rho = 0.84, delta rho = +0.12), follicle distribution pattern (kappa = 0.80, delta kappa = +0.21) and presence of a CL (kappa = 0.87, delta kappa = +0.05). No improvement was evident for FNPO (rho = 0.54, delta rho = -0.01). Both radiologists and reproductive endocrinologists demonstrated improvement in scores (p < 0.001).

**Conclusion:**

Reliability in evaluating ultrasonographic features of polycystic ovaries can be significantly improved following participation in a training workshop. If ultrasonographic evidence of polycystic ovaries is to be used as an objective measure in the diagnosis of PCOS, then standardized training modules should be implemented to unify the approach to evaluating polycystic ovarian morphology.

## Background

Diagnostic criteria and management procedures for polycystic ovary syndrome (PCOS) are highly controversial and hotly debated in the literature [1,2]. The use of ultrasonography in the diagnosis of PCOS is one such point of contention [3]. In 1990, the first attempt by experts to generate international consensus criteria for PCOS resulted in exclusion of polycystic ovaries as a potential marker of the syndrome [4]. While these criteria were heralded as a legitimate first step toward characterizing the clinical spectrum of PCOS, they did override standards and practices employed in the UK and most of Europe where the diagnosis had long been based on ultrasonography [3]. At the time, evidence of polycystic ovaries was considered "suggestive" and not diagnostic of PCOS since there were numerous reports of polycystic ovarian morphology in normal asymptomatic women (up to 30%) and in conditions other than PCOS such as normal to late puberty, hyperprolactinemia and congenital adrenal hyperplasia [5-9].

In the years that followed, it became apparent that polycystic ovaries were in fact, a consistent finding in women demonstrating biochemical and clinical evidence of PCOS [10-13]. Moreover, it was discovered that asymptomatic women with polycystic ovaries demonstrated subtle endocrine and metabolic abnormalities [3,6,11]. In 2003, ultrasonographic evidence of polycystic ovaries was incorporated as a third diagnostic marker of PCOS at a joint-meeting of the European Society for Human Reproduction and Embryology (ESHRE) and American Society for Reproductive Medicine (ASRM) [1]. Revisions to the consensus criteria were intended to broaden the clinical spectrum of PCOS and therefore allowed for a diagnosis based on identification of *two of three *criteria: 1) oligo- or chronic anovulation, 2) clinical and/or biochemical hyperandrogenism and 3) polycystic ovaries on ultrasonography. While there is concern that these criteria are too expansive [2], they do reflect majority opinion that polycystic ovaries are a significant component of PCOS.

The current ultrasound guidelines supported by ESHRE/ASRM define the polycystic ovary as having 12 or more follicles measuring 2 – 9 mm and/or an increased ovarian volume of >10 cm^3 ^[1]. Unlike previous definitions, no assessment of stromal echotexture or follicle distribution pattern is necessary [5]. The cutoff value for ovarian volume is based on cumulative reports of mean volumes >10 cm^3 ^for polycystic ovaries [14] while the cutoff value of 12 follicles *throughout the entire ovary *was shown to have 99% specificity and 75% sensitivity in distinguishing between polycystic and normal ovaries [15,16]. At present, the reproducibility of these values has not been reported nor has the level of variability associated with the evaluation of these criteria been established. We are aware of only one study in which observer variation in the ultrasound diagnosis of polycystic ovaries has been assessed [17]. Amer *et al*. showed that when the polycystic ovary was defined as having ≥ 10 follicles, an ovarian volume ≥ 12 cm^3 ^and a bright echogenic stroma, a diagnosis was agreed upon among observers only 51% of the time while observers agreed with themselves only 69% of the time [17]. Significant variability when making the diagnosis suggested that the criteria employed were either too subjective or too insensitive to allow for good agreement [14]. Unfortunately, the extent to which any of these features contributed to the subjectivity of the diagnosis was not evaluated.

We recently attempted to determine where variability in the ultrasound diagnosis might lie by determining the level of inter-observer variability associated with identifying and quantifying individual ultrasonographic features of polycystic ovaries (e.g., total follicle count, ovarian volume, etc.) [18]. In our previous study, overall agreement among radiologists and reproductive endocrinologists was only moderate to poor. We learned that observers varied significantly in their approach to analyzing each ultrasonographic feature and this accounted for discrepancies in agreement. Differences in technique were mostly related to differences in training among medical disciplines and learning institutions. The primary objective of the current study was to determine if a training workshop could reduce inter-observer variation when evaluating ultrasonographic features of polycystic ovaries. We hypothesized that agreement among observers could be vastly improved following the review of relevant Acoustic Physics principles, ovarian ultrasound image acquisition and interpretation, and a detailed analysis of ovarian structures by *in vitro *water bath scanning.

## Methods

### Study subjects

Thirty women diagnosed with PCOS by a single primary care provider were enrolled in the study. PCOS was defined as having 2 of 3 characteristics: 1) oligo-anovulation (menstrual cycles <21 or >35 days), 2) clinical and/or biochemical evidence of hyperandrogenism (modified Ferriman-Gallwey score > 6 [19] and/or a free androgen index ≥ 5 [20]), 3) polycystic ovaries on ultrasound (≥ 12 follicles measuring 2 – 9 mm in diameter or an ovarian volume > 10 cm^3^) [1]. Other etiologies of anovulatory infertility such as hyperprolactinemia, hypercortisolemia, thyroid dysfunction and 21-hydroxylase deficiency were excluded. Subjects had to range in age from 18 to 35 and could not have used hormonal contraception, fertility medications or valproate in the three months prior to enrolment. The ability to visualize at least one ovary by transvaginal ultrasonography was required for inclusion.

### Transvaginal ultrasonography

Study subjects underwent a single transvaginal ultrasound scan. In all subjects, including those reporting regular menstrual cycles, the scan occurred at a random time (during their menstrual cycle). Scans were performed by a single ultrasonographer using an UltraSonix RP ultrasound scanner equipped with a 9-MHz transvaginal probe (UltraSonix, Version 2.3.5, Vancouver, BC). Ovaries were scanned from the inner to outer margins in both the transverse and sagittal planes. All scans were recorded digitally. Digital video clips were subsequently transferred to a custom-designed database at the Women's Health Imaging Research Laboratory (WHIRL) at the University of Saskatchewan for post-hoc image analysis.

### Selection and randomization of ultrasonographic image clips

Digital video clips of 30 individual ovaries (one from each subject) were selected for analysis from the 60 ovaries scanned. Each ovary was designated an electronic folder yielding a total of 30 individual "polycystic ovary cases". Each folder contained 2 digital video clips and a still image of a single cross-section through the ovary. One video clip represented a sweep through the ovary in the transverse plane and the other represented a sweep in the sagittal plane. Links to these 30 folders were randomly generated for each of the 6 observers such that no observer reviewed the cases in the same order. Following the ultrasound workshop, links to these folders were reassigned for each observer ensuring that images were reviewed in a different order than the first.

### Evaluation of ultrasonographic records

Two staff radiologists, a senior Radiology resident (fifth and final year of residency program) and 3 clinician/scientists with training in Reproductive Endocrinology (a reproductive endocrinologist and 2 research scientists with training in transvaginal ultrasonography) reviewed the cases at WHIRL workstations for the following endpoints: 1) follicle number per ovary (FNPO), 2) follicle number per single cross-section (FNPS), 3) largest follicle diameter, 4) ovarian volume, 5) follicle distribution pattern and 6) presence of a corpus luteum (CL). For the FNPO endpoint, observers were asked to count the total number of follicles ≥ 2 mm in the entire ovary using only one of the video clips provided (i.e., clearly labeled "for FNPO"). For the FNPS endpoint, observers were asked to count the total number of follicles ≥ 2 mm in the single ovarian cross-section provided. Observers were instructed to use both video clips to select and measure the follicle with the largest diameter. Observers were also asked to judge for each clip (i.e., pattern in the transverse plane and pattern in the sagittal plane), whether follicles in the ovary were predominantly distributed in a "peripheral" pattern or whether follicles were distributed more heterogeneously ("even") throughout the stroma. In the event that neither category could best describe the follicle distribution pattern, a designation of "other" could be made. Observers were asked to calculate ovarian volume from measurements of the largest and widest diameters of the ovaries in the transverse and sagittal planes using the equation for a prolate spheroid [π/6 (anterposterior diameter^2 ^× transverse diameter)] [21]. Lastly, observers were instructed to use both video clips to determine the presence or absence of a corpus luteum. Two complementary software programs (FRAME^© ^and SYNERGYNE 2^©^, Saskatoon, SK, Canada) were used to analyze the digital images. Recordings could be viewed at any speed or direction including, frame-by-frame analysis. Colour/contrast adjustments and linear measurements could be made on any frame of the video clip.

### Ultrasound training workshop

**Figure 1:**
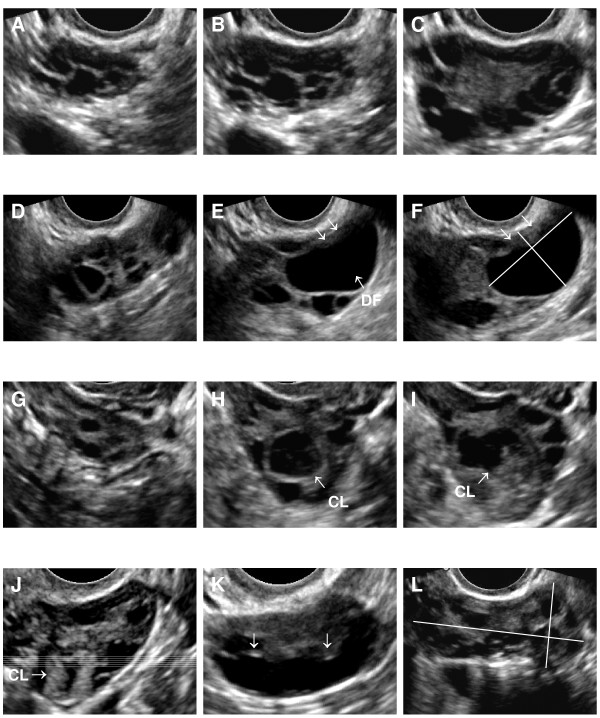
**Ultrasonographic image attributes encountered during the inter-observer evaluation of polycystic ovaries are presented**. Panels A, B and C represent sequential images through a polycystic ovary where an apparently 'even' distribution of follicles is evident at the end of the ovary while a peripheral distribution of follicles is evident at the central portion of the ovary (Panel C). Designation of follicle pattern was to be made at the largest cross-sectional image of the ovary. Panels D, E and F depict sequential images through a polycystic ovary containing a large dominant follicle (DF). An area of acoustic artifact (arrows) is present along the top portion of the dominant follicle closest to the transducer (Panels E and F). Both the follicle wall and area of acoustic artifact were to be included in the measurements of the largest follicle diameter (Panel F). Panels G, H and I demonstrate sequential images through a polycystic ovary containing a large cystic corpus luteum (CL). The cystic CL has a hyperechoic crenulated border and a flocculent fluid filled cavity. A polycystic ovary containing a CL with thick hyperechoic walls and a small fluid-filled cavity is presented in Panel J for comparison with Panel I. A polycystic ovary containing a concentrated collection of follicles along the lower margin of the ovary is presented in Panel K. The number of follicles counted would depend on the number of lobulations perceived among the "string of pearls". Areas of specular reflection (arrows) could be used to aid in the identification of some individual follicles. A transverse cross-section through an irregular shaped polycystic ovary is shown in Panel L. Estimations of the longest and widest orthogonal diameters of the ovary were to be made for estimations of ovarian volume.

Following the first evaluation of the case folders, observers participated in a 2-hour ultrasound workshop focused on Acoustic Physics and ovarian ultrasound image acquisition and interpretation. A detailed analysis of ovarian structures by *in vitro *water bath scanning and dissection of bovine ovaries was also performed (i.e. most appropriate animal model for humans) [22]. Discussion among observers with regard to their individual approach for assessing each of the ultrasound parameters resulted in a list of consensus instructions and tips for re-evaluating polycystic ovarian morphology (Table 1). Observers were given 10 days from the date of the ultrasound workshop to complete their re-evaluation of the case folders. Representative images discussed during the workshop are presented in Figure 1.

**Table 1:** Consensus instructions for assessing ultrasonographic features of polycystic ovaries.

**Counting ovarian follicles**
▪ When counting follicles throughout the entire ovary, scroll through the digital recording using the frame-by-frame feature.
▪ Count a single sweep through the ovary multiple times to generate a consensus count.
▪ When counting follicles in a single plane, adjust contrast to improve visibility/conspicuity of follicles.
▪ Most follicles will appear as round anechoic areas but some follicles, particularly, those atretic, will appear irregularly shaped or compressed.
▪ Consider that discernible walls may not be apparent between adjacent follicles.
▪ Use artifacts of specular reflection to aid in the identification of follicles.
▪ Do not count the cystic cavity of the corpus luteum as a follicle.

**Measuring the largest follicle diameter**
▪ Convince yourself by scrolling back and forth through the digital recoding that the follicle you have chosen is a single follicle.
▪ Freeze the recording at the frame which represents the largest cross-sectional area of the follicle in question.
▪ Adjust contrast and enlarge the image.
▪ The first linear measurement should be that of the largest longitudinal plane.
▪ Measurements should include the follicle wall as well as any area of acoustic artifact.
▪ The second linear measurement is that which bisects the first line at a right angle.
▪ The mean of these two lines represents the Largest Follicle Diameter.
▪ Make largest follicle measurements for both image clips and report the largest of the two.
▪ Do not count the corpus luteum as the largest follicle.

**Calculating ovarian volume and identifying follicle distribution pattern**
▪ Convince yourself by moving back and forth through the digital recording that you recognize the limits of the ovary.
▪ Freeze the recording at the frame which represents of the largest cross-sectional area.
▪ Adjust contrast and enlarge the image.
▪ Decide on the follicle distribution pattern in this cross-section (i.e. peripheral or even).
▪ If this image contains a follicle ≥ 10 mm or a large cystic CL, designate the follicle pattern as other.
▪ The first linear measurement should be that of the largest longitudinal plane.
▪ Draw your line keeping in mind that small round anechoic areas may lie outside the ovarian capsule (i.e. small vessels that should not be confused as follicles) and that an area of acoustic artifact may be present.
▪ The second linear measurement should represent the widest plane of the ovary and should cross the longitudinal measurement at a right angle.
▪ Repeats these measurements for the second clip and use the averages of the longitudinal and antero-posterior measurements to calculate volume.

**Identifying corpora lutea (CL)**
▪ CL may appear as a large cystic structure or a smaller hyperechoic structure with a small to negligible fluid-filled cavity.
▪ Walls of the CL are generally thick, hyperechoic and crenulated.
▪ The cystic cavity of CL generally has heterogeneous areas of echogenicity.
▪ Ensure that you can identify the CL in both sagittal and transverse planes before deciding on its presence.

### Ethical considerations

This study was approved by the University of Saskatchewan Biomedical Research Ethics Review Board. All study procedures conformed to the Canadian TriCouncil Guidelines for Human Research and International Good Clinical Practice Guidelines. Informed consent was obtained from all volunteers.

### Statistical analysis

Descriptive statistics (mean ± SEM and range) of clinical, hormonal and metabolic features of the study subjects were generated from clinical and laboratory medical records obtained at the time of evaluation for PCOS. Mean measurements (± SEM) for FNPO, FNPS, largest follicle diameter and ovarian volume were calculated and Tukey's multiple comparison tests were used to determine if differences among observers before and after the ultrasound workshop were significant. Lin's concordance correlation coefficients (ϱ) and kappa statistics for multiple raters (κ) were used to assess inter-observer agreement for continuous and discrete measures, respectively [23,24]. P and κ values that approximated 1 denoted perfect agreement while values that approximated 0 denoted agreement no better than that by chance. Guidelines for evaluating level of agreement were: >0.80 good, 0.60 – 0.80 moderate/fair, <0.60 poor [25]. These empirical guidelines are more stringent than the highly contested values originally reported by Landis and Koch [26]. Changes in agreement (Δϱ or Δκ) following the ultrasound workshop were calculated and Tukey's multiple comparison tests were used to determine significant changes in agreement following the ultrasound workshop.

## Results

### Study subject demographics

Clinical, hormonal and metabolic features of the study subjects are summarized in Table 2. Forty-seven percent (14/30) of volunteers were obese (>30 kg/m^2^), 10% (3/30) were overweight (26 – 30 kg/m^2^) and 43% (13/30) were lean (<25 kg/m^2^). Twenty-seven percent (8/30) reported menstrual cycles every 21 – 35 days, 33% (10/30) had cycles every 36 – 90 days and 40% (12/30) had cycles >90 days a part. Eighty-three percent (25/30) of subjects had hirsutism and 73% (22/30) had an elevated free androgen index. Ten percent of subjects (3/30) had no evidence of hirsutism or biochemical hyperandrogenemia. Seven percent (2/30) of subjects demonstrated impaired fasting glycemia while the remaining subjects had normal fasting glucose levels. Forty percent (12/30) of subjects were however, categorized as insulin resistant by an increased homeostatic model assessment of insulin resistance value.

**Table 2:** Clinical, hormonal and metabolic features of PCOS study subjects.

	**Mean ± SEM**	**Range**	**Normal Values**
**Age (yr)**	28.5 ± 0.7	19 – 35	-
**BMI (kg/m2)**	30.1 ± 1.4	19.4 – 45.0	20 – 25
**Waist Circumference (cm)**	95.3 ± 3.1	70.0 – 140.0	< 88
**Menstrual Cycle Length (d)**	106 ± 17	25 – 365	21 – 35
**mFG Score**	9.6 ± 1.1	0 – 24	< 6
**Testosterone (nmol/L)**	2.3 ± 0.2	0.75 – 5.0	< 2.5
**SHBG (nmoll/L)**	46.7 ± 4.2	13.0 – 97.2	18 – 114
**Free Androgen Index**	6.1 ± 0.7	1 – 19	< 5
**DHEA-S (μmol/L)**	5.1 ± 0.3	2.2 – 8.8	0.9 – 12.0
**17OH-Progesterone (nmol/L)**	3.8 ± 0.4	1.2 – 11.2	0.3 – 12.1
**LH:FSH Ratio**	2.3 ± 0.3	0.6 – 7.7	< 2
**Cortisol (nmol/L)**	303 ± 23	130 – 640	130 – 640
**Prolactin (μg/L)**	13.1 ± 0.9	4.4 – 20.0	3.0 – 20.0
**TSH (mIU/L)**	1.9 ± 0.2	0.5 – 4.8	0.3 – 5.5
**Fasting Glucose (mmol/L)**	4.9 ± 0.1	4.2 – 6.7	< 6.1
**Fasting Insulin (pmol/L)**	78.1 ± 9.4	21.0 – 185.0	< 100
**HOMA-IR**	3.0 ± 0.4	0.7 – 8.1	< 3

Normative values for the local health region are provided. Body mass index (BMI), modified Ferriman-Gallwey (mGF), sex hormone binding globulin (SHBG), dehydroepiandrosterone sulfate (DHEA-S), follicle stimulating hormone (FSH), luteinizing hormone (LH), thyroid stimulating hormone (TSH), homeostatic model assessment of insulin resistance (HOMA-IR).

### Overall inter-observer agreement

**Figure 2:**
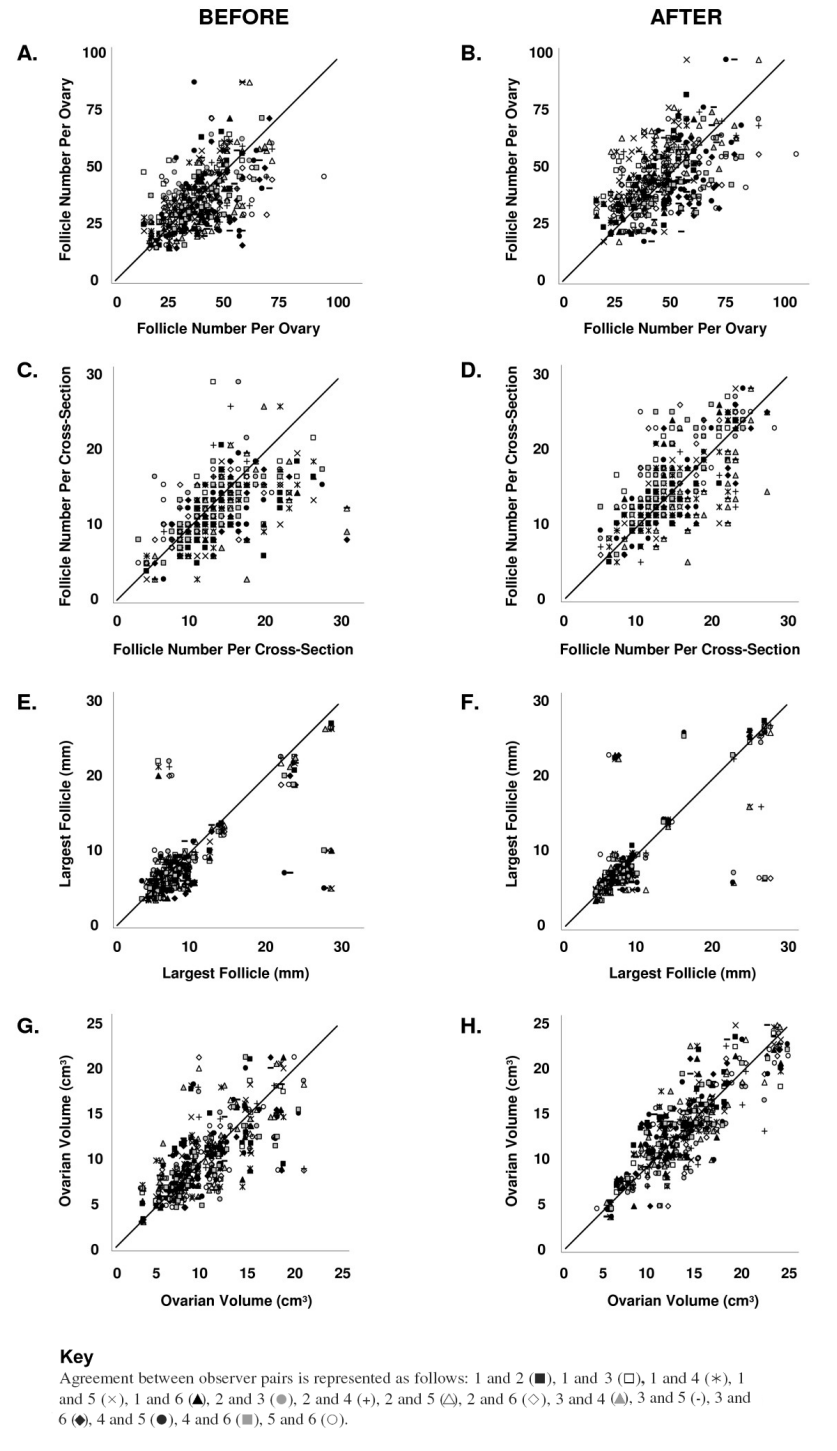
**Scatter plots of follicle number per ovary counts (FNPO; A and B), follicle number per single cross-section counts (FNPS; C and D), largest follicle diameter measurements (E and F) and ovarian volume calculations (G and H) by all possible pair-wise combinations of the six observers before and after the ultrasound workshop are presented**. Perfect agreement between two observers corresponds to a slope of 1 (diagonal line). Before the workshop, agreement was best for ovarian volume and largest follicle diameter and poorest for FNPO and FNPS. Following the workshop, visible improvement was apparent for FNPS, largest follicle diameter and ovarian volume. No improvement in FNPO was evident.

Scatter plots of pair-wise agreement in FNPO counts, FNPS counts, largest follicle diameter measurements and ovarian volume calculations by the six observers are presented in Figure 2. Perfect agreement corresponds to a slope of 1 (diagonal line). Before the ultrasound workshop, inter-observer agreement was best for ovarian volume and largest follicle diameter and poorest for FNPO and FNPS. Following the ultrasound workshop, visible improvement as judged by an increase in the number of points aggregating along the diagonal line was apparent in the assessment of FNPS (compare 2C and 2D), largest follicle diameter (compare 2E and 2F) and ovarian volume (compare 2G and 2H). No improvement in the evaluation of FNPO was evident (compare 2A and 2B).

Before the workshop, FNPO and FNPS counts were 35.6 ± 0.9 and 11.8 ± 0.3 follicles, respectively. Measurements of the largest follicle diameter were 7.8 ± 0.3 mm and ovarian volume measurements were 9.7 ± 0.3 cm^3^. Following the workshop, FNPO counts (42.9 ± 1.0 follicles, p < 0.0001), FNPS counts (14.3 ± 0.4 follicles, p < 0.0001) and ovarian volume measurements (13.1 ± 0.3 cm^3^, p < 0.0001) were higher than those reported before the workshop. By contrast, measurements of largest follicle diameter were similar to those made before the workshop (8.1 ± 0.4 mm, p = 0.09). Both before and after the ultrasound workshop, all cases fulfilled the criteria for the diagnosis of a polycystic ovary based on ESHRE/ASRM guidelines of ≥ 12 follicles per ovary. At no time did observers make counts lower than 12 follicles per ovary (even in the presence of a dominant follicle or CL). Measurements made by radiologists (RAD) and reproductive endocrinologists (REI) are presented in Table 3. Before the workshop, RAD and REI made similar measurements for FNPO and ovarian volume but not for FNPS (p = 0.0001) or largest follicle diameter (p = 0.002). Differences in FNPS and largest follicle measurements were resolved following the workshop although, RAD tended to make larger ovarian volume measurement post-workshop (p = 0.001).

**Table 3:** Ultrasonographic measurements made by radiologists and reproductive endocrinologists before and after an ultrasound workshop.

	**FNPO**		**FNPS**		**Largest Follicle (mm)**		**Ovarian Volume (cm3)**	
				
	**Before**	**After**	**Before**	**After**	**Before**	**After**	**Before**	**After**
**RAD**	34.5 ± 1.4a	42.8 ± 1.6b	11.0 ± 0.4c	14.4 ± 0.5d	7.1 ± 0.4f	8.1 ± 0.5g	9.7 ± 0.4h	13.5 ± 0.4i
**REI**	36.6 ± 1.1a	43.1 ± 1.3b	12.6 ± 0.5e	14.1 ± 0.5d	8.3 ± 0.5g	8.1 ± 0.5g	9.6 ± 0.4h	12.7 ± 0.4j

Different letters represent significant differences between groups (p > 0.05) both before and after the ultrasound workshop.

The overall level of pair-wise agreement among the six observers before and after the ultrasound workshop is summarized in Table 4. Before the workshop, agreement between pairs of observers ranged from 0.25 to 0.78 for the assessment of FNPO, 0.33 to 0.91 for FNPS, 0.37 to 0.96 for largest follicle diameter, 0.45 to 0.87 for ovarian volume, 0.69 to 0.93 for presence of a CL and 0.26 to 0.90 for follicle distribution pattern (Note: data were combined for pattern in the sagittal and transverse planes since no differences were detected). Overall inter-observer agreement for continuous and discrete measures was fair (ϱ = 0.63 and κ = 0.71, respectively). Following the workshop, agreement between pairs of observers increased for all parameters except FNPO. Post-workshop agreement ranged from 0.49 to 0.81 for FNPS (p = 0.01), 0.52 to 0.98 for largest follicle diameter (p = 0.13), 0.77 to 0.90 for ovarian volume (p = 0.0004), 0.76 to 0.97 for presence of a CL (p = 0.08) and 0.69 to 0.97 for follicle distribution (p = 0.0001). Overall inter-observer agreement for continuous and discrete measures was significantly improved (ϱ = 0.71; p = 0.0003 and κ = 0.84; p = 0.001).

**Table 4:** Level of pair-wise agreement for six observers assessing ultrasonographic features of polycystic ovaries before and after an ultrasound workshop.

	**Overall Concordance Correlation Coefficient (ρ)**					**Overall Kappa Statistic (κ)**		
		
	**FNPO**	**FNPS**	**Largest ** **Follicle**	**Ovarian** **Volume**	**Average**	**Corpus** **Luteum**	**Follicle** **Pattern**	**Average**
**BEFORE**	0.55	0.59	0.67	0.72	0.63	0.82	0.59	0.71
**AFTER**	0.54	0.70	0.77	0.84	0.71	0.87	0.80	0.84
**Δρ/Δκ**	-0.01	+0.11	+0.10	+0.12	+0.08	+0.05	+0.21	+0.13
**P-value**	0.73	0.01	0.13	0.0004	0.0003	0.08	0.0001	0.001

Agreement among RAD and REI assessing ultrasonographic features of polycystic ovaries before and after the ultrasound workshop are presented in Table 5. Before the workshop, RAD demonstrated better agreement in the evaluation of FNPS (p = 0.01) and distribution pattern (p = 0.01) while REI demonstrated better agreement in measurements of largest follicle diameter (p = 0.01). Following the workshop, differences when evaluating FNPS, largest follicle and distribution pattern were resolved. Overall agreement following the ultrasound workshop was similar among REI and RAD for continuous measures (ϱ = 0.71 vs. 0.71, p = 0.98). However, REI demonstrated better agreement for discrete measures (κ = 0.88 vs. 0.79, respectively; p = 0.01).

**Table 5:** Agreement among radiologists and reproductive endocrinologists assessing ultrasonographic features of polycystic ovaries before and after an ultrasound workshop.

	**FNPO**		**FNPS**		**Largest ** **Follicle**		**Ovarian ** **Volume**		**Corpus ** **Luteum**		**Follicle ** **Pattern**	
						
	**Before**	**After**	**Before**	**After**	**Before**	**After**	**Before**	**After**	**Before**	**After**	**Before**	**After**
**RAD**	0.55a	0.56a	0.71b	0.70b	0.55d	0.69d, e	0.66f	0.88f	0.84g	0.82g	0.68h	0.76h, i
**REI**	0.49a	0.50a	0.51c	0.65b	0.86e	0.85e	0.71f	0.83f	0.82g	0.93g	0.48j	0.83i

Different letters represent significant differences between groups (p > 0.05) both before and after the ultrasound workshop.

## Discussion

Our objective was to determine the effect of an ultrasound training workshop on the inter-observer variability associated with evaluating ultrasonographic features of polycystic ovaries. Our approach involved the assessment of transvaginal ultrasound recordings of 30 polycystic ovaries for six features, by six observers with training in either Radiology or Reproductive Endocrinology, both before and after an ultrasound workshop. The use of ultrasonographic recordings for the determination of inter-observer variability is a commonly used and highly feasible approach [17]. It involved volunteers undergoing only one endovaginal scan and avoided the presence of numerous observers in the ultrasound suite which may be considered intrusive or embarrassing for the study participant [17]. Furthermore, it mimicked practices in Radiology where digital images/recordings captured by trained sonographers are presented to radiologists for post-hoc evaluation. Had it been practical to have six observers perform their own scans, we suspect that differences in training, technique and experience at the time of image acquisition would have further compounded the level of variability reported among observers.

Observers were given minimal instructions prior to their initial analysis of the images in hopes that each observer would best use his or her own skill-set in the assessments. Agreement was initially poor for FNPO, FNPS and follicle distribution pattern, moderate for largest follicle diameter and ovarian volume, and good for identification of a CL. These findings were consistent with our previous study in which four observers analyzed transvaginal ultrasound recordings of polycystic ovaries for similar morphologic endpoints [18]. The results of our current study showed that inter-observer agreement could be significantly improved when observers participated in a workshop focused on evaluating ovarian morphology. Discussion among radiologists and reproductive endocrinologists which culminated in the formation of consensus guidelines for assessing ultrasonographic features was the primary factor responsible for improved agreement following the ultrasound workshop.

The current ESHRE/ASRM recommendations for the ultrasonographic evaluation of polycystic ovaries state that ultrasound scans (preferably, transvaginal) be performed during the early follicular phase (i.e., days 3 – 5) or three to five days following a hormonally-induced bleed in women with chronic anovulation [1]. This recommended time of ultrasonography corresponds to a time during the natural menstrual cycle in which follicle population is dramatically increasing, yet maximum follicle diameters are generally <10 mm [27]. Despite these recommendations, many women still present for ultrasonographic evaluation at random times during their menstrual cycle. We felt it instructive to mimic real-life situations by having participants present for their ultrasounds at random such that observers would encounter multiple follicle sizes and/or ovulation glands when assessing polycystic ovarian morphology. While we recognize that not all the ultrasonographic endpoints assessed are used to diagnose polycystic ovaries, each of these features is routinely evaluated at the time of ovarian ultrasonography since each gives important information regarding ovarian function/dysfunction.

Poor agreement in the evaluation of FNPO demonstrated by this current study was in contrast to past reports of very good inter-observer agreement in total antral follicle counts (2 – 10 mm) using real-time or stored 2D and 3D transvaginal ultrasonographic imaging [28,29]. In previous studies, good agreement among observers was associated with antral follicle counts in the order of approximately 10 follicles per ovary [28,29]. However, it should be noted that both groups also reported a distinct decrease in inter-observer agreement when follicle counts were greater than 15 [28,29]. In the present study, follicle counts in women diagnosed with PCOS by the ESHRE/ASRM criteria generally ranged from 35 – 40. That we were counting more than three times as many follicles per ovary may have accounted for differences in agreement between studies. That there were fewer follicles to count in a single cross-section (i.e., ~12 follicles), may have also accounted for the better agreement levels reported for FNPS compared to FNPO.

Difficulty in counting follicles lay in the high degree of crowding that occurred among adjacent follicles. In having performed an ultrasono-histopathological assessment of bovine ovaries, it was apparent that follicles could appear as either round or irregular in shape due to atresia or compression by adjacent structures. It was also evident that follicle clustering caused adjacent follicular walls to be imperceptible on ultrasonography. Discriminating among adjacent follicles would therefore, depend on the observer's perception of the number of lobulations present among a collection of cysts rather than the identification of septa between follicles. With these points in mind, significant improvement in the assessment of FNPS was evident following the ultrasound workshop. Improvement resulted even though mean FNPS counts were significantly higher than those reported before the ultrasound workshop. The higher follicle counts likely reflected an improved awareness of what actually constituted an ovarian follicle on ultrasonography.

An increase in mean follicle counts was also reported for the assessment of FNPO following the ultrasound workshop. However, unlike the FNPS endpoint, inter-observer agreement was not improved for FNPO. Failure to improve agreement in FNPO may be interpreted to mean that the level of subjectivity associated with counting follicles throughout the entire ovary is insurmountable. The current ultrasound recommendations argue that the ability to reliably count 12 follicles is sufficient to ensure an accurate diagnosis. Our study supports the notion that multiple observers can agree on counting at least 12 follicles per ovary. However, there is merging evidence that total follicle population relates to degree of symptomology and therefore, health risks for women with PCOS [30]. Ascertaining the clinical relevance of discrete aspects of ovarian morphology may therefore help identify persons at risk for PCOS and/or progression of the syndrome.

Differences in ovarian volume measurements before the ultrasound workshop were related to differences in measurement technique among observers. Some observers would measure the widest and longest orthogonal planes of the ovary while others would measure the longest plane first and then draw their width measurement such that it bisected the longitudinal plane at a right angle (i.e., this may or may not have represented the widest plane of the ovary). After agreeing to uniformly measure only the longest and widest orthogonal planes, ovarian volume measurements were significantly greater following the workshop and inter-observer agreement for ovarian volume proved excellent. This was consistent with several studies reporting good inter-observer agreement in the ultrasound assessment of ovarian volume [31-34]. The subjectivity associated with counting follicles may be interpreted to suggest that calculation of ovarian volume should represent the primary method of diagnosing polycystic ovaries. Unfortunately, there are limitations to determining ovarian volume that must be acknowledged. For example, accurate measurements of ovarian volume can only be made during the early follicular phase when there is generally no dominant follicle (>10 mm) or cystic CL to overestimate the size of the ovary. Cutoff levels for increased ovarian volume in polycystic ovaries are debatable since there is significant overlap with the normal population [35]. Also, it is important to note that not all polycystic ovaries will be enlarged despite demonstrating a grossly elevated follicle count [14]. More importantly, there is no universally accepted method of calculating ovarian volume [14]. In this study, we employed the equation for a prolate spheroid to calculate ovarian volume rather than the equation for a prolate ellipsoid which is recommended by the ESHRE/ASRM consensus. The equation for a prolate spheroid was found to correlate better with volume measurements of polycystic ovaries made by 3D-ultrasonography than the formula for a prolate ellipsoid [21].

The most improvement in agreement was seen for the evaluation of follicle distribution pattern. Before the workshop, many observers expressed clear frustration and reluctance to assign a distribution pattern since digital sweeps through the ovary would often show both even and peripheral follicle distribution patterns depending on what portion of the ovary was represented. After discussion, it was concluded that the designation follicle pattern should occur at the single largest cross-sectional view of the ovary in keeping with previous definitions of polycystic ovarian morphology [5]. That is, observers would now scroll to the digital frame that represented the largest cross-sectional area of the ovary and decide on both follicle pattern and measurements of the widest and longest diameters of the ovary (i.e., for measurement of ovarian volume) using that individual frame. It was decided that in instances where a preovulatory follicle or a cystic CL was present in the largest plane a designation of 'other' be made. Inter-observer agreement for evaluation of follicle distribution pattern became exceptional following this consensus approach.

The presence of an ovulation gland is a highly important finding to report in women with PCOS given its implications for fertility and risk of endometrial hyperplasia. While CL are typically identifiable during the luteal phase, it is important to recognize that CL, albeit non-functional, may be visualized ultrasonographically during the early follicular phase coinciding with the recommended time for evaluation of PCOS [36]. Following the ultrasound workshop, agreement in the identification of a CL was good among virtually all pairs of observers. Disagreement among observers was typically noted only when a CL appeared as a cystic structure versus when it appeared as a hyperechoic structure with a small to negligible fluid-filled cavity [36]. In these instances, there was a tendency to mistake a cystic CL for a dominant preovulatory follicle. Mistaking a CL for a large follicle also accounted for outlier measurements of the largest follicle diameter. Clues recognized by the observers as being helpful in distinguishing between CL and preovulatory follicles included the presence of a floccuent cystic cavity and/or crenulated hyperechoic postovulatory follicular walls which are apparent only in CL. Had observers performed their own ultrasound scans, the use of Doppler in real-time could have facilitated the identification of CL and likely improved agreement among observers for this endpoint [36].

In summary, variability in the ultrasound diagnosis of polycystic ovaries likely reflects poor to moderate inter-observer agreement when identifying and quantifying individual characteristics of polycystic ovaries. Agreement in assessing ultrasonographic features of polycystic ovaries can be significantly improved when evaluators generate consensus guidelines for assessing ultrasonographic endpoints. Our study supports the notion that standardized training modules for characterizing polycystic ovarian morphology are needed if ultrasonographic evidence of polycystic ovaries is to be used as an objective measure in the diagnosis of PCOS. Also, that collaboration and communication among imaging specialists in different medical disciplines is necessary for generating a truly consensual approach. Developing reliable and unified methods for the acquisition and interpretation of ultrasonographic images of polycystic ovaries is critical for ensuring the timely identification and intervention of PCOS.

## Competing interests

The authors declare that they have no competing interests.

## Authors' contributions

MEL conceived, designed and coordinated the study, performed the ultrasound scans, conducted the statistical analyses and drafted the final manuscript. DRC clinically evaluated the study volunteers for PCOS. DRC, AKP, SK, DAL, TGB and MEL participated in the ultrasound workshop and generated consensus criteria for the sonographic evaluation of polycystic ovaries. AKP aided in the statistical analyses and helped draft the manuscript. RAP participated in the conception and design of the study and provided resources and equipment to complete the study. All authors read and approved the final manuscript.
